# Long term outcome of combined phacoemulsification and excisional goniotomy with the Kahook Dual Blade in different subtypes of glaucoma

**DOI:** 10.1038/s41598-021-90223-5

**Published:** 2021-05-21

**Authors:** Ahmed Al Habash, Abdulrahman Albuainain

**Affiliations:** 1 Department of Ophthalmology, King Fahd Hospital of the University, College of Medicine, Imam Abdulrahman bin Faisal University, Dammam, Kingdom of Saudi Arabia; 2 Eye and Laser Centre, Bahrain Defence Force Hospital, Royal Medical Services, Riffa, Kingdom of Bahrain

**Keywords:** Medical research, Outcomes research, Lens diseases, Diseases, Eye diseases, Glaucoma

## Abstract

To characterize changes in intraocular pressure (IOP) and IOP-lowering medications through up to 2 years of follow-up in patients undergoing combined phacoemulsification and excisional goniotomy with the Kahook Dual Blade (phaco-KDB), with simultaneous goniosynechialysis in cases of angle-closure glaucoma. Prospective, non-comparative, interventional case series. Consecutive patients with medically-treated glaucoma and visually-significant cataract underwent combined surgery. Analysis was conducted on open-angle (OAG) and angle-closure (ACG) glaucoma groups separately. Thirty-seven patients with OAG (24 with primary OAG and 13 with pseudoexfoliation glaucoma) and 11 with ACG were enrolled. In OAG eyes, mean (standard error) baseline IOP was 21.1 (0.9) mmHg and through 24 months of follow-up was reduced by 6.4–7.7 mmHg (24.6–32.1%; p ≤ 0.0001 at all time points). In ACG eyes, mean baseline IOP was 20.8 (1.6) mmHg and was reduced by 6.1–8.77 mmHg (23.4–39.0%; p ≤ 0.0353). Mean medications were reduced by 61.9–89.1% (p ≤ 0.0001) in OAG eyes and by 56.3–87.3% (p ≤ 0.0004) in ACG eyes. Phaco-KDB significantly lowered IOP ~ 30% and medications by > 50% through 24 months. This combined procedure provides meaningful long-term reductions in IOP and need for IOP-lowering medication and does not adversely affect visual rehabilitation in eyes with cataract and glaucoma.

## Introduction

Cataract is the world's most common cause of blindness^1^, and glaucoma represents an important cause of blindness worldwide as well^2^. Effective and safe intraocular pressure (IOP)-lowering treatments appropriate for patients with all types and severities of disease would help to halt progression of glaucomatous optic nerve damage and subsequent decline in quality of life^3^. Conventional surgical techniques for the treatment of glaucoma typically provide greater IOP reductions than more conservative medical and laser therapies^4,5^, but trabeculectomy and tube shunts have a higher risk of vision-threatening complications, including early postoperative complications such as hypotony and lifetime risk of bleb or device-related complications^6,7^. Longer visual recovery times, activity limitations, need for frequent follow-up, and secondary office-based or surgical interventions in the early postoperative phase also compromise the patient's quality of life as well as healthcare costs.

In recent years, a series of novel and less-invasive surgical techniques have been developed to provide meaningful IOP reductions with lower risk of complications compared to conventional glaucoma surgery. Most of these procedures avoid the formation of a filtering bleb—and its complications—by shunting aqueous humor across the obstructed trabecular meshwork (TM) into Schlemm's canal (SC) or into the suprachoroidal space, although a few techniques rely on subconjunctival filtration^8,9^. These procedures are considered safe and effective options that can be combined with cataract extraction and may prevent or delay the need for more invasive and higher-risk filtering or shunt surgeries, especially when used in early or moderate stages of the disease^10,11^.

The Kahook Dual Blade (KDB; New World Medical, Rancho Cucamonga, CA, USA) is an ophthalmic knife which is used to perform surgical ab interno trabeculectomy (commonly referred to as excisional goniotomy or gonioectomy)^12^. Since the development of the KDB in 2015, a rising number of studies have established its efficacy and safety in reducing IOP and medication burden^13,14^. Unlike conventional goniotomy, which is frequently implemented in congenital glaucoma, the KDB's design allows complete resection of diseased TM on the inner wall of SC, allowing the flow of aqueous from the anterior chamber to the distal outflow system^12^. The KDB also has a favorable safety profile^13,14^. The most common complication is intraoperative or early postoperative blood reflux that is to be expected with the unroofing of several collector channels and is generally transient^15^.

The newer glaucoma procedures are most commonly utilized in mild-to-moderate glaucoma, due to moderate efficacy compared to subconjunctival filtering procedures^8–11^. Therefore, most studies of these procedures have been limited to these populations. However, a previous study on stand-alone KDB goniotomy has shown promising efficacy and safety in severe glaucoma patients^15^. Another study demonstrated the efficacy of KDB combined with phacoemulsification (Phaco-KDB) in glaucoma patients across the spectrum of disease severity, of whom 22 had severe glaucoma^14^. To our knowledge, no study has specifically examined the long-term efficacy of KDB goniotomy combined with cataract surgery in patients with different types of glaucoma in our region of the world. In this study, we describe long-term (up to 36 months) outcomes of phacoemulsification and excisional goniotomy using the KDB, combined with goniosynechialysis in cases of angle-closure glaucoma, in patients with cataract and different types and stages of glaucoma.

## Methods

### Study design

This was a prospective, non-comparative, uncontrolled, non-randomized interventional case series of consecutive patients undergoing combined Phaco-KDB, with simultaneous goniosynechialysis in cases of angle closure glaucoma. All the surgeries were performed by a single surgeon (A.H.) at King Fahd Hospital of the University, Dammam, Saudi Arabia over the course of 2 years. The protocol was reviewed and approved by Imam Abdulrahman Bin Faisal University IRB. Approval was given on the understanding that the "Guidelines for Ethical Research Practice" were adhered to, and all patients provided written informed consent to participate. Participating patients were adults 18 years or older with medically-managed glaucoma and visually significant cataract undergoing Phaco-KDB for reduction of IOP and/or medication burden, combined with goniosynechialysis in cases of angle-closure glaucoma. Patients undergoing any other combined procedures, active uveitis, coexisting retinopathy that limits visual acuity potential, active neovascularization, angle dysgenesis and those with less than 12 months of follow-up, were excluded. IOP was measured by Goldmann tonometry twice, once each by a glaucoma specialist and a glaucoma fellow, at each visit; the mean of these two measurements represented the IOP at that visit for purposes of analysis.

### Surgical technique

The combined Phaco-KDB procedure has been previously described^9,11^. Briefly, following standard phacoemulsification and intraocular lens implantation, the anterior chamber was filled with ophthalmic viscosurgical device (OVD). The KDB was inserted into the anterior chamber and under intraoperative gonioscopy advanced to the nasal TM. In eyes with angle-closure glaucoma, goniosynechialysis was performed first, as described by Dorairaj^16,17^. The KDB’s pointed tip engaged the peripheral iris at the base of each peripheral anterior synechia (PAS) and dissected the PAS with gentle radial pressure within the iris plane toward the pupillary center to reveal the trabecular meshwork. The excisional goniotomy was then performed as previously described^13,14^. The instrument’s tip engaged TM until the heel of the device rested within SC. The blade was then advanced along the TM, which became elevated and stretched as it was guided up the ramp to the two parallel cutting blades that removed an intact TM strip. Using the dip and strip technique in which the TM is punctured with the KDB at one end of the intended excision, the KDB then entered TM at the opposite end of the intended excision and was advanced to the first puncture site. The KDB was then removed from the eye and the excised strip of TM removed from the eye with forceps.

### Statistical analysis

Data collected in this study included baseline demographic information as well as visual acuity (VA), IOP, and IOP-lowering medications at every time point. Intraoperative and postoperative adverse events were also recorded. Postoperative data were collected at Day 1, Weeks 2, 4 and 6, and Months 2–3, 6, 9, 12, 18, 24 post-surgery. VA was best-corrected VA (BCVA) preoperatively and beginning 4–6 weeks postoperatively. IOP was measured with Goldmann applanation tonometry. In determining the number of IOP-lowering medicines used at each time point, combination products were counted by the number of constituents and oral carbonic anhydrase inhibitors were also included in the count. The co-primary outcomes of this analysis were the reductions of both IOP and IOP-lowering medications from baseline at each postoperative time point. These outcomes were assessed using paired *t* tests. Secondary outcomes included change in BCVA from baseline (also assessed using paired-tests), as well as the proportion of patients with > 20% IOP reduction, with IOP < 18 mmHg and < 15 mmHg, with > 1 medication reduction, and medication-free at each time point beginning at Month 2–3 (after postoperative stabilization). These analyses were conducted separately in eyes with open-angle and angle-closure glaucoma due to differences in mechanisms of these two subtypes. No specific hypotheses were tested and formal power and sample size calculations were not undertaken. The level of significance was taken to be 0.05. Means are reported with standard errors. Data were analyzed using SAS version 9.4 (SAS Institute Inc, Cary, NC).

## Results

Data from 48 eyes of 48 subjects were analyzed. Demographic and baseline data are given in Table 1. Among 37 subjects with OAG (24 with POAG and 13 with pseudoexfoliation glaucoma) who underwent phaco-KDB, the mean (standard error) age was 65.4 (1.6) years and most (64.9%) were male. Among 11 subjects with ACG who underwent phaco-KDB combined with goniosynechialysis, the mean age was 60.4 (2.8) years and most (72.7%) were female. The mean follow-up time was 25.6 (1.3) months in eyes with OAG and 26.2 (2.2) months in eyes with ACG.

**Table 1:** Demographics and baseline glaucoma status in 48 eyes of 48 subjects.

Parameter	OAG N = 37	ACG N = 11
Age (yr), mean (SE)	65.4 (1.6)	60.4 (2.8)
**Gender, n (%)**		
Male	24 (64.9)	3 (27.3)
Female	13 (35.1)	8 (72.7)
Follow-up (months), mean (SE)	25.6 (1.3)	26.2 (2.2)
**Operative eye, n (%)**		
Right	22 (59.5)	9 (81.8)
Left	15 (40.5)	2 (18.2)
Cup-disc ratio, mean (SE)	0.65 (0.03)	0.74 (0.05)

*ACG* angle-closure glaucoma, *OAG* open-angle glaucoma (includes primary and pseudoexfoliation open-angle glaucoma), *SE* standard error.

### IOP outcomes

**Figure 1:**
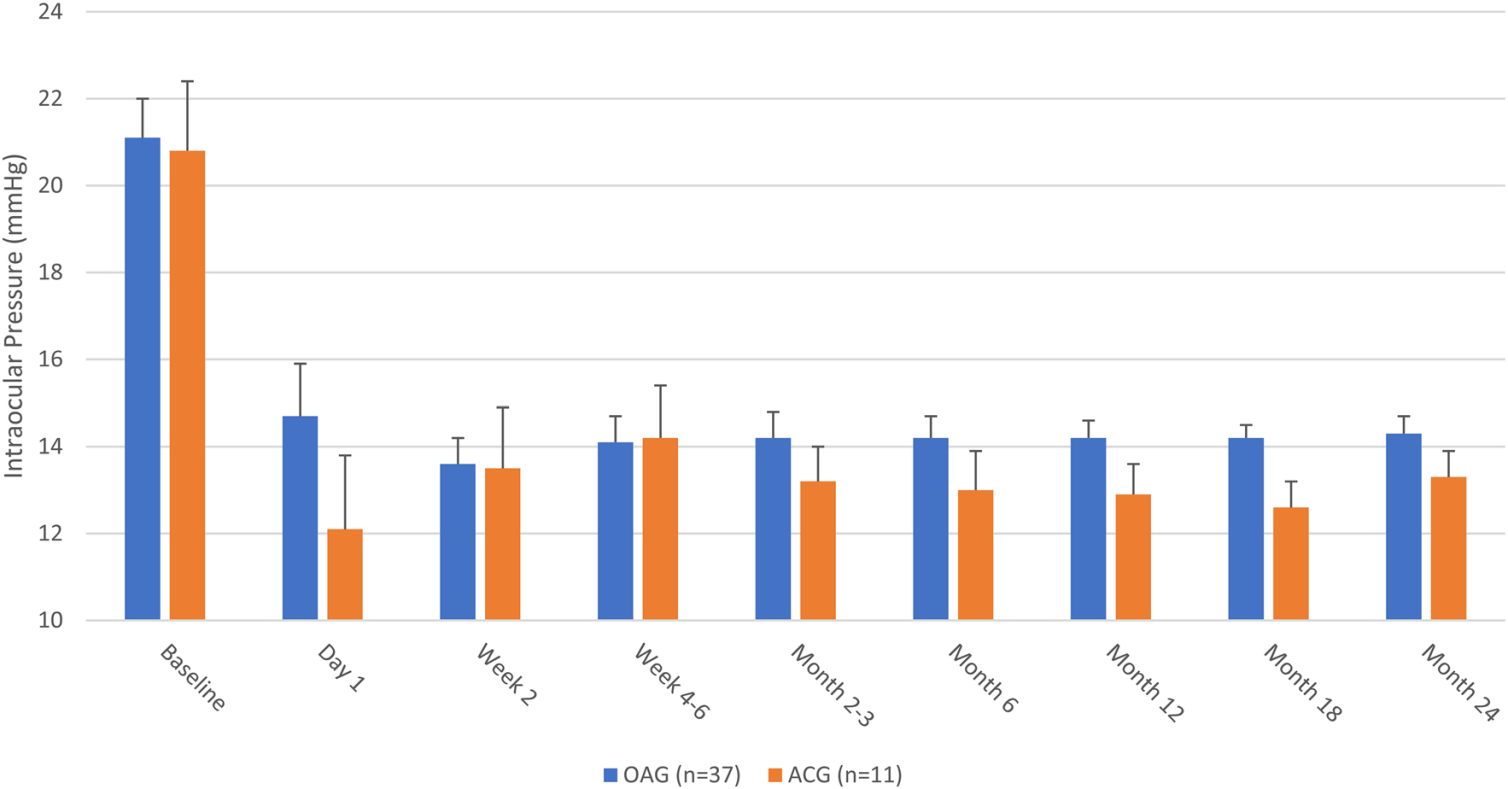
Mean IOP at each visit in eyes with open-angle glaucoma (n = 37) and angle-closure glaucoma (n = 11). In eyes with OAG, p ≤ 0.0001 at all time points; in eyes with ACG, p ≤ 0.0353 at all time points. Error bars represent standard error.

IOP data at each time point for the OAG group are given in Table 2 and Fig. 1. Among eyes with OAG, baseline IOP was 21.1 (0.9) mmHg and through up to 2 years of follow-up ranged from 13.6–14.7 mmHg, representing absolute IOP reductions of 6.4–7.7 mmHg and relative reductions of 24.6–32.1% (p ≤ 0.0001 at all time points). At Month 24 (when 30/37 eyes were seen [81.1%]), mean IOP was 14.1 mmHg (a reduction of 7.7 mmHg [32.1%]; p < 0.0001). Across all time points, IOP reductions of ≥ 20% were achieved by 73.0–83.3% of eyes, IOP ≤ 18 mmHg by 89.2–97.3% of eyes, and IOP ≤ 15 mmHg by 72.9–83.9% of eyes; at Month 24, these secondary endpoints were achieved by 83.3%, 96.7%, and 73.3% of eyes, respectively (Table 4).

**Table 2:** Intraocular pressure, medication, and visual acuity data at each time point in eyes with open-angle glaucoma (n = 37).

	Baseline	Day 1	Week 2	Week 4–6	Month 2–3	Month 6	Month 12	Month 18	Month 24
Number of eyes	37	37	37	32	37	37	37	32	30
Mean (SE) IOP, mmHg	21.1 (0.9)	14.7 (1.2)	13.6 (0.6)	14.1 (0.6)	14.2 (0.6)	14.2 (0.5)	14.2 (0.4)	14.3 (0.3)	14.1 (0.4)
Mean (SE) IOP change from baseline, mmHg	–	− 6.7 (1.5)	− 7.5 (1.0)	− 6.4 (1.0)	− 6.8 (1.0)	− 6.9 (1.0)	− 6.9 (0.9)	− 6.9 (0.9)	− 7.7 (0.8)
Mean (SE) % IOP change from baseline	–	− 24.6 (7.3)	− 29.4 (6.0)	− 26.2 (4.8)	− 27.7 (4.4)	− 27.1 (4.7)	− 26.9 (4.8)	− 28.0 (4.1)	− 32.1 (3.7)
p (IOP mean change from baseline)	–	0.0001	< 0.0001	< 0.0001	< 0.0001	< 0.0001	< 0.0001	< 0.0001	< 0.0001
Mean (SE) medications, n	3.3 (0.2)	–	0.4 (0.2)	0.8 (0.2)	1.1 (0.2)	1.2 (0.2)	1.5 (0.2)	1.5 (0.3)	1.5 (0.3)
Mean (SE) medication change from baseline, n	–	–	− 2.9 (0.2)	− 2.5 (0.2)	− 2.2 (0.2)	− 2.1 (0.2)	− 1.8 (0.2)	− 1.9 (0.2)	− 1.9 (0.2)
Mean (SE) % medication change from baseline	–	–	− 89.1 (3.9)	− 79.1 (4.8)	− 71.1 (4.7)	− 68.4 (4.9)	− 61.9 (5.8)	− 61.9 (6.2)	− 62.7 (6.5)
P (medication mean change from baseline)	–	–	< 0.0001	< 0.0001	< 0.0001	< 0.0001	< 0.0001	< 0.0001	< 0.0001
Mean (SE) BCVA, logMAR	1.17 (0.16)	1.14 (0.17)	0.66 (0.11)	0.42 (0.11)	0.34 (0.07)	0.27 (0.05)	0.27 (0.05)	0.23 (0.04)	0.22 (0.04)
Mean (SE) BCVA change from baseline, logMAR	–	− 0.03 (0.16)	− 0.52 (0.13)	− 0.59 (0.09)	− 0.83 (0.12)	− 0.90 (0.13)	− 0.90 (0.13)	− 0.86 (0.14)	− 0.82 (0.14)
Mean (SE) % BCVA change from baseline, logMAR	–	65.70 (49.92)	− 25.11 (14.61)	− 63.08 (6.71)	− 71.28 (5.04)	− 76.60 (4.47)	− 75.02 (4.69)	− 77.13 (5.05)	− 76.70 (5.42)
P (BCVA mean change from baseline)	–	0.8682	0.0002	< 0.0001	< 0.0001	< 0.0001	< 0.0001	< 0.0001	< 0.0001

*BCVA* best-corrected visual acuity, *IOP* intraocular pressure, *logMAR* logarithm of the minimum angle of resolution, *mmHg* millimeters of mercury, *SE* standard error.

IOP data at each time point for the ACG group are given in Table 3 and Fig. 1. Among eyes with ACG, baseline IOP was 20.8 (1.6) mmHg and through up to 2 years of follow-up ranged from 12.1–14.2 mmHg, representing absolute IOP reductions of 6.1–8.77 mmHg and relative reductions of 23.4–39.0% (p ≤ 0.0353 at all time points). At Month 24 (when 10/11 eyes were seen [90.1%]), mean IOP was 13.3 mmHg (a reduction of 7.9 mmHg [31.4%]; p = 0.0060). Across all time points, IOP reductions of ≥ 20% were achieved by 70.0–81.8% of eyes, IOP ≤ 18 mmHg by 90.9–100% of eyes, and IOP ≤ 15 mmHg by 70.0–90.9% of eyes; at Month 24, these secondary endpoints were achieved by 70%, 100%, and 70% of eyes, respectively (Table 4).

**Table 3:** Intraocular pressure, medication, and visual acuity data at each time point in eyes with angle-closure glaucoma (n = 11).

	Baseline	Day 1	Week 2	Week 4–6	Month 2–3	Month 6	Month 12	Month 18	Month 24
Number of eyes	11	11	11	10	11	11	11	10	10
Mean (SE) IOP, mmHg	20.8 (1.6)	12.1 (1.7)	13.5 (1.4)	14.2 (1.2)	13.2 (0.8)	13.0 (0.9)	12.9 (0.7)	12.6 (0.6)	13.3 (0.6)
Mean (SE) IOP change from baseline, mmHg	–	− 8.7 (2.2)	− 7.4 (2.4)	− 6.1 (2.5)	− 7.6 (1.8)	− 7.8 (2.2)	− 7.9 (2.0)	− 8.6 (1.9)	− 7.9 (2.2)
Mean (SE) % IOP change from baseline	–	− 39.0 (9.1)	− 30.3 (9.2)	− 23.4 (11.3)	− 32.6 (7.6)	− 31.7 (9.5)	− 33.3 (7.1)	− 36.8 (6.1)	− 31.4 (8.8)
p (IOP mean change from baseline)	–	0.0026	0.0111	0.0353	0.0014	0.0051	0.0024	0.0012	0.0060
Mean (SE) medications, n	3.7 (0.3)	–	0.6 (0.3)	0.8 (0.3)	1.3 (0.4)	1.3 (0.4)	1.6 (0.4)	1.8 (0.4)	1.8 (0.4)
Mean (SE) medication change from baseline, n	–	–	− 3.2 (0.4)	− 2.8 (0.4)	− 2.5 (0.4)	− 2.5 (0.4)	− 2.1 (0.3)	− 2.1 (0.4)	− 2.1 (0.4)
Mean (SE) % medication change from baseline	–	–	− 87.3 (6.6)	− 78.3 (7.5)	− 68.5 (9.0)	− 69.4 (8.9)	− 60.3 (9.1)	− 56.3 (9.1)	− 56.3 (9.1)
P (medication mean change from baseline)	–	–	< 0.0001	< 0.0001	0.0001	< 0.0001	0.0001	0.0004	0.0004
Mean (SE) BCVA, logMAR	0.63 (0.17)	1.07 (0.32)	0.45 (0.08)	0.34 (0.09)	0.31 (0.09)	0.23 (0.07)	0.18 (0.06)	0.20 (0.07)	0.19 (0.07)
Mean (SE) BCVA change from baseline, logMAR	–	0.45 (0.25)	− 0.18 (0.13)	− 0.15 (0.15)	− 0.31 (0.13)	− 0.39 (0.13)	− 0.45 (0.12)	− 0.46 (0.13)	− 0.47 (0.13)
Mean (SE) % BCVA change from baseline, logMAR	–	117.2 (79.42)	0.93 (21.29)	− 0.21 (31.76)	− 36.28 (18.75)	− 58.29 (10.13)	− 70.82 (6.06)	− 70.32 (6.88)	− 71.12 (6.69)
P (BCVA mean change from baseline)	–	0.1062	0.2080	0.3354	0.0343	0.0123	0.0049	0.0073	0.0068

*BCVA* best-corrected visual acuity, *IOP* intraocular pressure, *logMAR* logarithm of the minimum angle of resolution, *mmHg* millimeters of mercury, *SE* standard error.

**Table 4:** Pre-specified IOP and medication outcomes at each time point for the open-angle glaucoma and angle-closure glaucoma groups.

	Month 2–3		Month 6		Month 12		Month 18		Month 24	
Glaucoma type	OAG	ACG	OAG	ACG	OAG	ACG	OAG	ACG	OAG	ACG
Number of eyes (n)	37	11	37	11	37	11	32	10	30	10
Percent achieving IOP reduction ≥ 20% compared to baseline	73%	81.8%	73%	81.8%	78.4%	72.7%	78.1%	80%	83.3%	70%
Percent achieving IOP ≤ 18 mmHg	89.2%	100%	91.9%	90.9%	97.3%	100%	96.9%	100%	96.7%	100%
Percent achieving IOP ≤ 15 mmHg	78.4%	81.8%	83.85	81.8%	72.9%	90.9%	81.3%	90%	73.3%	70%
Percent using ≥ 1 fewer medication compared to baseline	100%	100%	100%	100%	94.6%	100%	96.7%	100%	81.8%	100%
Percent medication-free	45.9%	36.4%	43.2%	36.4%	40.5%	27.3%	40.6%	20%	43.3%	20%

*ACG* angle-closure glaucoma, *IOP* intraocular pressure, *mmHg* millimeters of mercury, *OAG* open-angle glaucoma (includes primary and pseudoexfoliation open-angle glaucoma).

### Medication outcomes

**Figure 2:**
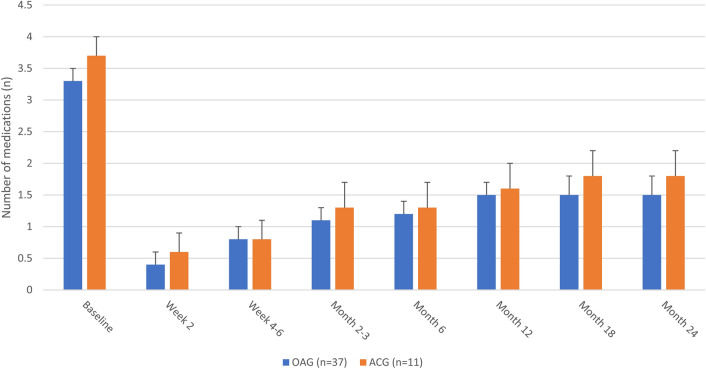
Mean number of IOP-lowering medications at each visit by glaucoma subtype. Error bars represent standard error. p < 0.0001 at all-time points in the full data set of all eyes (n = 48).

IOP medication data at each time point for the OAG group are given in Table 2 and Fig. 2. Among eyes with OAG, the mean number of IOP medications at baseline was 3.3 (0.2) and through up to 2 years of follow-up ranged from 0.4–1.5, representing absolute reductions of 1.8–2.9 medications and relative reductions of 61.9–89.1% (p ≤ 0.0001 at all time points). At Month 24, the mean number of medications was 1.5 (a reduction of 1.9 [62.7%]; p < 0.0001). Across all time points, 81.8–100% of eyes were using fewer medications than at baseline and 40.5–45.9% were medication-free; at Month 24, these secondary endpoints were achieved by 81.8% and 43.3% of eyes, respectively (Table 4).

IOP medication data at each time point for the ACG group are given in Table 3 and Fig. 2. Among eyes with ACG, the mean number of IOP medications at baseline was 3.7 (0.3) and through up to 2 years of follow-up ranged from 0.6–1.8, representing absolute reductions of 1.8–2.9 medications and relative reductions of 56.3–87.3% (p ≤ 0.0004 at all time points). At Month 24, the mean number of medications was 1.8 (a reduction of 2.1 [56.3%]; p = 0.0004). Across all time points, 100% of eyes were using fewer medications than at baseline and 20.0–36.4% were medication-free; at Month 24, these secondary endpoints were achieved by 100% and 20% of eyes, respectively (Table 4).

### Visual acuity outcomes

Visual acuity data at each time point are given in Table 2. Mean logMAR BCVA was 0.97 (0.11) at baseline and was significantly improved (p < 0.0005 at all-time points after Day 1) through 24 months of follow-up. At Months 24, mean BCVA was 0.20 (0.03). All eyes had improved or stable BCVA at last follow-up.

### Safety outcomes

The combined procedure was safe and well tolerated. Six eyes (12.5%) developed transient hyphema that resolved spontaneously in all cases, and 1 eye (2.1%) developed elevated IOP on the first postoperative day attributed to retained ophthalmic viscosurgical device which also resolved spontaneously. No eyes required any secondary surgical interventions for IOP control throughout the follow-up period.

## Discussion

In 2015, The Kahook Dual Blade (KDB, New World Medical, Rancho Cucamonga, CA) was launched in the United States. The KDB is a novel goniotomy blade produced to create a more complete removal of TM through a minimally invasive technique without any adjacent tissue injury^12^. The design of KDB has several key features to achieve a complete goniotomy. The tip is sharp, the heel fits comfortably within SC which allows smooth advancement of the blade without any collateral injury, the ramp of the blade stretches TM gently while blade advancement and the dual blades create parallel incisions facilitating excision of a strip of TM^12,18^. An additional benefit of the KDB is that it is a single-use, disposable instrument that does not require any additional special surgical equipment, without implant-related risks as no implant is left behind with this procedure^18^.

Reducing IOP or the medication burden are two main indications for combining glaucoma surgery with elective cataract surgery (as in most cases of POAG or pseudoexfoliation glaucoma) or in more urgent cases (as in acute ACG). Our prospective study is a real-world study that reveals the long experience of a single surgeon performing combined phaco-KDB demonstrates the safety, efficacy with a significant and persistent reduction in the IOP and the need for IOP-lowering medication throughout 24 months.

The IOP reductions observed in this study are consistent with IOP reductions reported in other studies of phaco-KDB in POAG eyes (12–27%)^13,14,19–26^. Similarly, medication reductions in the current study are similar to previously reported outcomes in POAG eyes (21–71%)^13,14,19–26^. These prior benchmarks were reported in studies generally of 6–12 months’ duration. The current study included data from all 48 subjects through 12 months and from 40/48 (83.3%) through 24 months. Throughout follow-up, IOP reductions remained stable, while medication reductions diminished somewhat in both OAG and ACG eyes. However, both IOP and medication reductions were significant from baseline at 24 months in both groups. Two previous prospective studies have been achieved. In multicenter interventional case series, Greenwood and colleagues evaluated 71 eyes undergoing phacoemulsification with goniotomy^14^. At 6 months, 58.3% of patients had at least a 20% reduction of IOP from baseline, and 61.7% were using at least one fewer IOP lowering medication. Similar results were observed in a subsequent prospective interventional case series of 52 patients undergoing phaco-KDB. At 12 months, a 26.3% reduction of IOP was observed in addition to a 50% reduction in the number of medications used^13^.

In addition to its efficient IOP lowering and medication reduction, KDB goniotomy furthermore shows a well-tolerated and safe profile. Overall, most complications were transient hyphemia with spontaneous resolution in only six eyes, and one eye with transient high IOP due to retained OVD as we usually intend to leave some OVD in the end of the surgery, which also resolved spontaneously and were non-sight threatening. These results correspond with prior reports in the literature^13–15,20,26^.

Earlier studies have hypothesized that angle procedures targeting the TM may be more effective among the pseudoexfoliation glaucoma patient population as the pseudoexfoliative material may be obstructing TM outflow^19^. Only one prior study to date has compared the success rates of KDB goniotomy between POAG and PXFG. In their study, Sieck et al. reported a higher success rate among PXFG (84.6%) compared to POAG (66.0%). However, this difference did not reach statistical significance after^19^. We pooled eyes with POAF and PXFG after determining that outcomes were not dissimilar between them (data not shown).

Strengths of this study include its length of follow-up and its patient population of Saudi Arabian glaucoma patients. Also, we have separately analyzed outcomes in the various glaucoma subgroups, which has not been reported in most prior studies with mixed-glaucoma samples. The lack of a control group—common to many retrospective analyses of novel glaucoma procedure outcomes—is a limitation that precludes benchmarking our results to other procedures in a head-to-head fashion. Our study design also precludes isolating the effects of phacoemulsification alone on IOP. Phacoemulsification alone can transiently lower both IOP and the need for medications in eyes with glaucoma. In a recent meta-analysis, mean IOP reductions of 15% and mean medication reductions of 0.38 were reported at 24 months^27^. In a separate report, the endurance of IOP reduction following phacoemulsification in eyes with PXFG was reported to be no more than 1 year^28^. This magnitude of effect is inadequate to explain the findings of our study, supporting the beneficial additive effect of combined phaco-KDB.

## Conclusions

In summary, Phaco-KDB significantly improved VA, lowered IOP ~ 25–30%, and lowered medications by > 50% through 24 months. This combined procedure provides meaningful long-term reductions in IOP and need for IOP-lowering medication and does not adversely affect visual rehabilitation in eyes with cataract and glaucoma.

## Data Availability

The datasets analyzed during the current study are available from the corresponding author on reasonable request.
